# A Pilot Study of Whether the Cold-Heat Syndrome Type is Associated with Treatment Response and Immune Status in Patients with Non-Small Cell Lung Cancer

**DOI:** 10.1155/2021/9920469

**Published:** 2021-06-22

**Authors:** Yujin Choi, Ojin Kwon, Chang-Min Choi, Mi-Kyung Jeong

**Affiliations:** ^1^Clinical Medicine Division, Korea Institute of Oriental Medicine, Daejeon 34054, Republic of Korea; ^2^Department of Pulmonary and Critical Care Medicine, Asan Medical Center, University of Ulsan College of Medicine, Seoul 05505, Republic of Korea

## Abstract

The cold-heat syndrome type (ZHENG) is one of the essential elements of syndrome differentiation in East Asian Medicine. This pilot study aimed to explore the characteristics of non-small cell lung cancer (NSCLC) patients treated with immune checkpoint inhibitors (ICIs) based on the cold-heat syndrome type. Twenty NSCLC patients treated with ICI monotherapy were included in the study and completed the cold-heat syndrome differentiation questionnaire. Demographic and clinical characteristics of the included patients were obtained through electronic medical records. Additionally, blood samples of 10 patients were analyzed with cytokine level and immune profiling. Patients were divided into two groups of cold type (*n* = 9) and non-cold type (*n* = 11), according to the cold symptoms questionnaire's cutoff point. No significant difference between the two groups was observed in clinical response to ICIs (*p*=0.668). Progression-free survival (PFS) seemed to be longer in patients with non-cold type than cold type (*p*=0.332). In patients with adenocarcinoma, the non-cold type showed longer PFS than the cold type (*p*=0.036). Also, there were more patients with PD-L1 negative in the cold type compared to the non-cold type (*p*=0.050). In immune profiling, the proportion of effector memory CD8 T-cells was higher in patients with cold type than with non-cold type (*p*=0.015), and the proportion of terminal effector CD8 T-cells was lower in patients with cold type than with non-cold type (*p*=0.005). This pilot study has shown the potential for differences in prognosis and immune status between patients with cold and non-cold types. Hopefully, it provides essential information and insight into NSCLC patients' characteristics from the perspective of syndrome differentiation. Further large-scale observational studies and intervention studies are required.

## 1. Introduction

Lung cancer is the leading cause of cancer death, and according to the 2018 global cancer statistics, it accounts for 18.4% of all cancer death [1]. Non-small cell lung cancer (NSCLC) is the most common type of lung cancer and accounts for over 85% of all lung cancer cases [2]. Recently, cancer immunotherapy has been developed targeting immune escape mechanisms exploited by cancer [3]. Progression of tumor cells is associated with immune suppression and dysregulating immune checkpoint protein by tumors [4, 5]. Immune checkpoint modulators, including program death-1 (PD-1) and program death-ligand 1(PD-L1) inhibitors targeting tumor-mediated immune tolerance, showed promising therapeutic outcomes by reversing cancer immunosuppression [6, 7]. Several anti-PD-1/PD-L1 antibodies were approved for patients with NSCLC, including the anti-PD-1 of nivolumab and pembrolizumab and the anti-PD-L1 of atezolizumab [8].

Complementary and alternative therapies including herbal medicine are used for patients with NSCLC to enhance effects of conventional cancer therapies and reduce adverse effects [9–11]. With recent development of immune checkpoint inhibitors (ICIs), clinical evidence of the combination therapy of herbal medicine with ICIs is limited. Still, considering herbal medicine can stimulate the function of various immune cells [8, 12, 13] by regulating the tumor immunosuppressive microenvironment [14, 15], a synergic effect between East Asian Medicine and ICIs is expected for cancer patients. Applying these treatments requires a holistic approach through “syndrome differentiation” [16, 17] in the perspective of yin-yang balance [18, 19].

Syndrome differentiation, also named as pattern identification and “ZHENG,” is the representative characteristic of traditional medicine in Asian countries, including traditional Chinese medicine (TCM), traditional Korean medicine (TKM), and Kampo medicine. Practitioners comprehensively obtain clinical information by observation, listening, questioning, and pulse analysis and make a clinical decision of appropriate individualized therapy based on the syndrome differentiation of individuals [16, 20]. In previous clinical trials of herbal medicine for NSCLC, Chinese herbal regimens were chosen from the three prescriptions based on the TCM syndrome differentiation [9]. From the systematic review of *Astragalus*-containing herbal formulae combined with platinum-based chemotherapy for NSCLC, the addition of herbal formulae to chemotherapy was more effective when the herbal formulae were given based on the syndrome differentiation [21]. Syndrome differentiation also plays an important role in predicting prognosis and treatment responses in cancer patients. Related research studies have been conducted. In cancer patients, more severe yin-deficiency symptoms are related to shorter survival rate [22]. In another report of advanced lung adenocarcinoma patients, gefitinib has shown better effect on patients with cold syndrome than on patients with heat syndrome [23].

For the fundamental information for further studies, we planned to explore the clinical characteristic and immune function based on the “ZHENG” in NSCLC patients treated with ICIs. Cold versus heat syndrome type differentiation is the important and essential element of syndrome differentiation in East Asian Medicine [24–27]. Also, a reliable and validated questionnaire of cold-heat syndrome type differentiation is available [28]. In this pilot study, we aimed to explore the clinical response and immune status of NSCLC patients treated with ICI monotherapy, based on the cold-heat syndrome differentiation.

## 2. Materials and Methods

### 2.1. Study Design and Participants

This study is a pilot study conducted in one hospital in Korea, including 20 patients with NSCLC undergoing monotherapy of ICIs. Inclusion criteria were as follows: (1) patients who diagnosis as stage IIIB or stage IV of NSCLC histologically or cytologically; (2) patients who are undergoing monotherapy of ICIs (pembrolizumab, nivolumab, and atezolizumab); and (3) patients whose ECOG performance status was 0 to 2. Eligible patients who agreed to participate with written informed consent filled out the questionnaires about the syndrome differentiation. Demographic and clinical characteristics of the included patients were obtained by the electrical medical record. Of the total 20 patients, 10 patients with cold or non-cold type were selected, and the immune profiles of patients' blood samples were additionally analyzed. The protocol was approved by the Institutional Review Board of the Asan Medical Center (approval number: 2018-0755).

### 2.2. Cold-Heat Syndrome Type Differentiation

A cold-heat syndrome differentiation questionnaire based on usual symptoms was used to measure the score of cold type and heat type [28]. The questionnaire is composed of eight items of cold type symptoms and seven items of hot type symptoms. Each item score ranges from 1 to 5, and the total score of cold type ranges from 8 to 40, while that of heat type ranges from 7 to 35. The cutoff point for cold type and non-cold type is reported to be 23.5, and that for heat type and non-heat type is reported to be 20.5 in the above reference.

By the cold syndrome questionnaire, there were 9 patients with cold type, and 11 patients with non-cold type. In the case of heat syndrome differentiation, there were 5 patients with heat type, and 15 patients with non-heat type. The demographic, clinical, and tumor characteristics of patients were compared in patients with cold type and in patients with non-cold type, according to the total score of the cold syndrome questionnaire.

### 2.3. Immune Profiling Assay

Blood samples of 10 patients were additionally analyzed for measuring cytokines level and immune cell profiling. We selected 10 patients that could represent the cold type and non-cold type based on the cold-heat syndrome type differentiation questionnaire. Five patients representing the cold type were selected based on their high cold syndrome scores. Five patients representing the non-cold type were selected based on their low cold syndrome scores and heat syndrome scores. CorPlex™ Human cytokine panel (Quanterix Corporation, Lexington) was used to measure the following cytokines in patients' blood: interferon-gamma (IFN-*γ*), IL-1*β*, IL-4, IL-5, IL-8, IL-10, IL-12p70, IL-22, and tumor necrosis factor-alpha (TNF*α*) [29, 30]. Ten T-cell cytokines were selected to measure tumor-associated immune response. IL-2, IL-12p70, IFN-*γ*, and TNF*α* are representative T helper 1 (Th1) cell-associated cytokines and IL-4, IL-5, IL-6, and IL-10 are T helper 2 (Th2) cell-related cytokines [31]. Also, an immune cell profile was obtained by Maxpar Direct Immune Profiling Assay (Fluidigm Corporation, San Francisco) [32]. The proportion of each immune cell's population frequencies by total intact live cells was calculated.

### 2.4. Statistical Analysis

Continuous variables were presented as mean ± standard deviation, and categorical variables were presented as frequency (%). Comparing proportions between two groups was carried out by the chi-square test or Fisher's exact test. Comparing means between two groups was carried out by an independent t-test. The software used for all the statistical analyses was R version 4.0.4 [33].

### 2.5. Cluster Analysis

The scores of each item in the cold-heat syndrome differentiation questionnaire were entered into the cluster analysis, and the 20 patients were divided into two groups. Hierarchical clustering was done using the R package factoextra [34, 35]. After standardizing the data, a dissimilarity matrix was computed by the Euclidean method, and a hierarchical tree was created by linkage methods. The hierarchical tree was cut into two groups and visualized by a scatter plot. The mean standardized score of each item in two groups was presented in a radar chart, using the R package ggiraphExtra [36].

## 3. Results

### 3.1. Characteristics of NSCLC Patients with Cold Type and Non-Cold Type

From 22 August to 11 November 2020, 20 patients were included and completed the questionnaire about cold-heat syndrome differentiation. Based on the score of the cold syndrome, 11 (55%) patients were non-cold type, and 9 (45%) patients were cold type. The histologic type of included patients was divided into adenocarcinoma (*n* = 14) and squamous cell carcinoma (*n* = 6), and the characteristics of total patients and patients with adenocarcinoma were presented (Table 1). Mean ages were 61.4 ± 12.1 in the non-cold type, and 61.1 ± 8.9 in the cold type, and all three female patients were cold type. Patients in the non-cold type seemed to include more current smokers, compared to patients in the cold type, which was not statistically significant (*p*=0.141). Between non-cold type and cold type, difference in drug use was observed (*p*=0.047); atezolizumab was prescribed in 77.8% of patients in cold type, versus 27.3% in patients in non-cold type. Line of therapy was investigated, and a majority of patients in non-cold type applied ICIs as 2nd line therapy (81.8% in total patients, and 85.7% in patients with adenocarcinoma). There was no significant difference in clinical response between two groups (*p*=0.668 in total patients, *p*=0.549 in patients with adenocarcinoma). Progression-free survival seemed to be longer in patients with non-cold type (102.0 (43.0; 172.5) days), compared to that in patients with cold type (44.0 (42.0; 76.0) days), which was not statistically significant in total patients (*p*=0.332). In patients with adenocarcinoma, progression-free survival was longer in patients with non-cold type (130.0 (74.0; 166.0) than that in patients with cold type (43.0 (42.0; 45.0) days) (*p*=0.036).

PD-L1 status was evaluated by SP263 (Ventana Medical Systems, Tucson, AZ) and 22CS (Agilent Technologies, Santa Clara, CA) [37,38] in all patients, except for one patient. Expression of PD-L1 was categorized as negative (0%); greater than 0% and less than 50%; and greater than 50%. In total patients, there was a significant difference in PD-L1 status between the two groups (*p*=0.050). In patients with adenocarcinoma, PD-L1 negative seemed to frequently appear in cold type compared to non-cold type, which was not statistically significant (*p*=0.110). Proportion of mutated EGFR seemed to be higher in patients with cold type than in patients with non-cold type, although the difference between the two was not statistically significant (*p*=0.115). There was no difference observed between the two groups in ALK and KRAS status.

### 3.2. Immune Profiling of NSCLC Patients with Cold Type and Non-Cold Type

**Figure 1:**
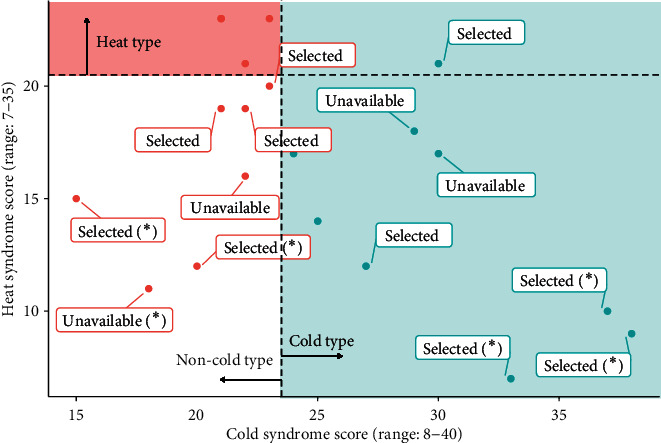
Cold syndrome and heat syndrome scores of 20 non-small cell lung cancer patients treated with immune checkpoint inhibitors. Ten patients representing cold types and non-cold type were selected for the immune profiling assay (selected). There were patients who were considered to be representative types, but not able to carry out immune profiling assay due to lack of samples (unavailable). In particular, six patients as representatives of cold type or non-cold type are indicated (*∗*).

**Figure 2:**
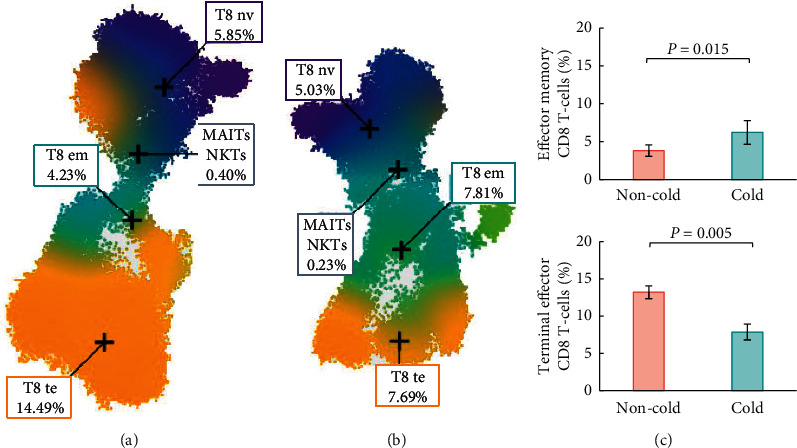
Representative CD8 T-cell region of Cen-se maps in (a) a patient with non-cold type, and (b) a patient with cold type. (c) The mean and standard error of effector memory and terminal effector CD8 T-cell percentage in 10 patients' blood sample. The proportion of effector memory CD8 T-cells (T8 em, the blue region in Figures 2(a) and 2(b)) was higher in patients with cold type than that in patients with non-cold type. Meanwhile, the proportion of terminal effector CD8 T-cells (T8 te, the yellow region in Figures 2(a) and 2(b)) was higher in patients with non-cold type than that in patients with cold type.

Based on cold and heat syndrome scores of patients, five representative cold types and five representative non-cold type patients were selected for immune profiling assay. Results of cold syndrome and heat syndrome scores of 20 patients and selected 10 patients for additional assay are presented in Figure 1. The level of cytokines and immune profiling of 10 patients' blood samples are shown in Table 2. There were some missing values for the level of cytokines because of the sample below the quantification limit. Patients with cold type seemed to have higher levels of cytokine IL-6 and IL-8 than patients with non-cold type, which was not statistically significant (*p*=0.208, *p*=0.208, respectively). In immune profiling, which was calculated as percent by intact live cells, effector memory CD8 T-cells were 3.8 ± 0.8% in patients with non-cold type and 6.2 ± 1.6% in patients with cold type (*p*=0.015). Also, terminal effector CD8 T-cells were 13.2 ± 1.9% in patients with non-cold type and 7.9 ± 2.4% in patients with cold type (*p*=0.005) (Figure 2). In the other immune profiles, there was no significant difference between the two groups.

### 3.3. Characteristics of NSCLC Patients Classified by Cluster Analysis

**Figure 3:**
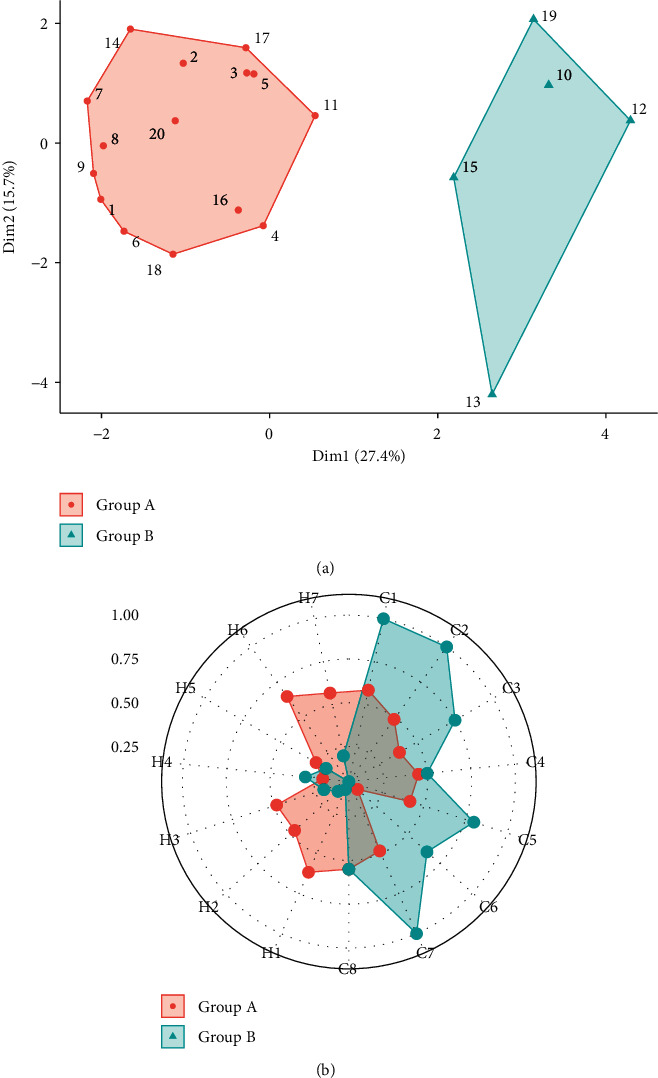
(a) Cluster plot of 20 patients classified by the symptoms of cold-heat syndrome, and (b) radar chart of mean standardized score of each symptom (eight symptoms in cold syndrome (ZHENG) are presented as C1–C8, and seven symptoms in heat syndrome (ZHENG) are presented as (H1–H7) in two cluster groups).

By hierarchical clustering using the score of symptoms in the cold-heat syndrome differentiation questionnaire, 20 patients were divided into group A (*n* = 15) and group B (*n* = 5) (Table 3, Table S3, and Figure 3(a)). All patients with non-cold type were included in group A, and some patients with cold type were included in group B. There were distinctive differences between group A and group B in their preferences for cold and heat: items of “I have an aversion to cold,” “I need to stay very warm,” “I like cool feeling,” and “I don't like hot or warm atmosphere” (*p* < 0.001, *p* < 0.001, *p* < 0.001, and *p*=0.047, respectively). It seems that there are more patients with PD-L1 expression higher than 50% in group A, compared to group B (*p*=0.051). Radar charts of the mean standardized score of each symptom (eight symptoms in cold syndrome are presented as C1–C8, and seven symptoms in heat syndrome are presented as H1–H7), in the two cluster groups are shown in Figure 3(b).

## 4. Discussion

In this pilot study, tumor-related characteristics and immune profiles of NSCLC patients treated with ICI monotherapy, depending on cold-heat syndrome type differentiation, were examined. According to the cold syndrome questionnaire, patients were divided into the cold type group and non-cold type group. Though the clinical response to ICI monotherapy was not different between the two groups, the progression-free survival periods seem to be longer in the non-cold type group than in the cold type group. Also, PD-L1 negative patients seem to be frequently observed in the cold type group, compared to the non-cold type group. In immune profiling, patients with cold type showed a higher proportion of effector memory CD8 T-cells and lower terminal effector CD8 T-cells than patients with non-cold type.

Patients were divided into the cold type or non-cold type according to the score of the cold syndrome questionnaire. By the questionnaire of cold-heat syndrome differentiation, patients can be divided into four types: no cold-heat, cold, heat, and cold-heat complex [39]. Of the total 20 patients, there were 7 patients with no cold-heat type; 8 patients with cold type; 4 patients with heat type; and 1 patient with cold-heat complex type. It was a pilot study with small sample size, and we divided patients into two groups with non-cold or cold type. A previous report suggested that excessive yin type, which is close to cold type, as an immunosuppressive status [40]. The results of comparing cold type versus heat type and non-heat type versus heat type are presented as Tables S1 and S2, respectively. In a previous study, 310 NSCLC patients were divided into yin-cold or yang-heat syndrome types by the diagnosis of two investigators using classical diagnostic processes for TCM [41]. Non-cold type corresponds to yang-heat syndrome type and cold type to yin-cold syndrome type. The proportion of male to female and proportion of smokers to nonsmoker were higher in yang-heat syndrome type than that in yin-cold syndrome type. This tendency was also observed in our pilot study: more male and current smoking patients in non-cold type than that in cold type. Different EGFR status between yin-cold and yang-heat syndrome types was also found in our result; the proportion of mutated EGFR tends to be higher in patients with cold type. EGFR mutations are generally associated with low response rates to ICIs in previous research studies [42, 43]. A systematic review has shown that ICIs are not beneficial for patients with EGFR mutant tumors [44]. Also, lung cancer cell expressing mutated EGFR is associated with decreased immune cell infiltration and T-cell mediated antitumor immunity [45]. Therefore, it can be estimated that the cold type patients with high EGFR mutation ratio may exhibit immune suppression and avoidance tendency [40].

There were more patients with PD-L1 negative in the cold type group compared to the non-cold type group. PD-L1 expression is one of the representative biomarkers for anti-PD-L1 or anti-PD-1 therapy [46]. In a clinical trial of pembrolizumab for the treatment of NSCLC, PD-L1 expression over 50% was correlated with the improved efficacy of pembrolizumab [47]. In this pilot study, three (27.3%) of 11 non-cold patients and one (11.1%) of nine cold type patients showed partial response to ICI treatment. The results of this pilot study are not enough to conclude that cold type is associated with low response to ICI treatment. Thus, further large-scale studies are needed. Also, interleukin 6 (IL-6) is another biomarker that has been reported to be associated with the prognosis of cancer patients. In previous studies, serum IL-6 level has shown an inverse correlation with the survival of cancer patients [48]. In this study, patients with cold type seemed to have a higher level of serum IL-6, IL-8, and IL-10 compared to that in patients with non-cold type, which was not statistically significant. Cytokines IL6 and IL-10 showing relatively high levels in patients with cold type are Th2-related cytokines [31, 49]. Th2 lymphocytes are associated with inhibition of the immune system [50]. Patients with lung cancer show increased systemic Th2 cytokine levels compared to healthy subjects [51]. These results also support the hypothesis that the cold type is associated with immune suppressive status.

Meanwhile, patients with cold type showed a higher proportion of effector memory CD8 T-cells and lower terminal effector CD8 T-cells, compared to patients with non-cold type. ICI therapy enhances the effector function of T-cells by the restoration of the activity of exhausted CD8 effector [52]. Circulating T-cell subpopulations were reported to correlate with immune response, and a high ratio of central memory to the effector T-cell in blood was correlated with increased tumor PD-L1 expression and longer progression-free survival in melanoma and NSCLC patients [53]. However, another study reported that in melanoma patients, higher peripheral CD8 effector memory T-cells were correlated with the clinical outcome [54]. In this study, it seemed that patients with cold type had a lower proportion of active CD8 T-cells than patients with non-cold type, which refers to immunosuppressive status [40]. Various biomarkers for checkpoint inhibitor immunotherapy, including tumor mutation burden, PD-L1 expression, T-cell-inflamed microenvironment, gut microbial diversity, and peripheral immune-based biomarker, have been suggested. Still, no definitive findings have yet been reported [46, 55, 56]. Although there was no significant difference in clinical response between the two groups, the progression-free survival periods seemed to be longer in the non-cold type group than in the cold type group. Consideration of the cold-heat syndrome type differentiation of patients could help provide additional information for understanding patients' characteristics and prognosis.

Cluster analysis using the score of each item in the cold-heat syndrome differentiation questionnaire was additionally conducted. Both items in cold syndrome and heat syndrome were included for the analysis. Patients in one group showed higher scores in heat syndrome symptoms, whereas patients in the other group showed higher scores in cold syndrome symptoms. The difference in symptoms of “intolerance to cold” was commonly observed by the cluster analysis of this pilot study and a previous study [41]. The signs of “yellow/thick tongue coating” and “red tongue” were also presented to be different in two groups in previous clustering [41]. In this study, only symptoms reported by patients were obtained, and information about tongue or pulse was not recorded. The classical East Asian medicine syndrome differentiation (ZHENG) includes inspection, auscultation, inquiry, and pulse [16], so further research efforts are required to analyze patients' objective signs, which can be obtained from practitioners or device.

To the best of our knowledge, this pilot study is the first study to investigate the clinical response and immune status of NSCLC patients treated with ICI monotherapy from the perspective of cold-heat syndrome differentiation. Hopefully, the results of this pilot study will provide essential information about the characteristics of NSCLC patients from the standpoint of syndrome differentiation in East Asian medicine. Also, there are several limitations to our study. First, the sample size of this study was relatively small. The aim of this pilot study was to obtain basic data for future research studies. Large-scale further research studies are needed to reach clinically and statistically meaningful conclusions. Second, we used the cold syndrome questionnaire's cutoff score, which was developed in a healthy population. Third, there were only three female patients in our study, and all female patients were included in the cold type group. Some differences between cold type and non-cold type groups were observed, but we could not exclude the possibility that they were caused by gender differences. In a previous study of cold and non-cold types, 38.1% of males and 61.9% of females corresponded to cold type, and 59.4% of males and 30.6% of females corresponded to non-cold type [28]. It seems that gender difference exists in distinguishing cold from non-cold type. Fourth, syndrome differentiation was done by the questionnaire, which only collects the subjective symptoms from the patients. In further studies, objective signs, which can be obtained by practitioners or medical devices, should be examined.

## 5. Conclusions

This pilot study has shown the potential differences in prognosis and immune status between patients with cold type and non-cold type. From the result of the study, NSCLC patients with cold type seem to present shorter progression-free survival and to have a low level of PD-L1 expression, compared to patients with non-cold type. Also, the proportions of effector memory and terminal effector CD8 T-cells were different between the two groups. When planning a clinical trial of combining herbal medicine and ICIs, the fundamental clinical information from our pilot study will hopefully provide useful insights into the patients' characteristics regarding cold-heat syndrome type (ZHENG). Further large-scale observational studies and intervention studies are required.

## Figures and Tables

**Table 1:** Demographic and clinical characteristics of 20 patients with non-cold or cold type.

	Total (*n* = 20)			Adenocarcinoma (*n* = 14)		
	Non-cold (*n* = 11)	Cold (*n* = 9)	*p* value	Non-cold (*n* = 7)	Cold (*n* = 7)	*p* value
Age (year)	61.4 ± 12.1	61.1 ± 8.9	0.959	58.1 ± 13.2	59.3 ± 7.5	0.846

Sex	—	—	0.148	—	—	0.193
Female	0 (0.0%)	3 (33.3%)		0 (0.0%)	3 (42.9%)	
Male	11 (100.0%)	6 (66.7%)		7 (100.0%)	4 (57.1%)	

Smoking status	—	—	0.141	—	—	0.214
Current	8 (72.7%)	3 (33.3%)		5 (71.4%)	2 (28.6%)	
Ex-smoker	2 (18.2%)	2 (22.2%)		1 (14.3%)	1 (14.3%)	
Nonsmoker	1 (9.1%)	4 (44.4%)		1 (14.3%)	4 (57.1%)	

Histologic type	—	—	0.844	—	—	
Adenocarcinoma	7 (63.6%)	7 (77.8%)		7 (100.0%)	7 (100.0%)	
Squamous	4 (36.4%)	2 (22.2%)		—	—	

Drug	—	—	0.047	—	—	0.091
Pembrolizumab	4 (36.4%)	2 (22.2%)		4 (57.1%)	1 (14.3%)	
Nivolumab	4 (36.4%)	0 (0.0%)		1 (14.3%)	0 (0.0%)	
Atezolizumab	3 (27.3%)	7 (77.8%)		2 (28.6%)	6 (85.7%)	

Line of therapy	—	—	0.059	—	—	0.013
2nd line therapy	9 (81.8%)	2 (22.2%)		6 (85.7%)	0 (0.0%)	
3rd line therapy	1 (9.1%)	2 (22.2%)		0 (0.0%)	2 (28.6%)	
4th line therapy	1 (9.1%)	4 (44.4%)		1 (14.3%)	4 (57.1%)	
7th line therapy	0 (0.0%)	1 (11.1%)		0 (0.0%)	1 (14.3%)	

No. of cycle^a^	7.0 ± 5.7	3.3 ± 1.1	0.110	7.0 ± 4.3	3.0 ± 1.2	0.106
Ongoing	3 (27.3%)	2 (22.2%)		2 (28.6%)	2 (28.6%)	

Clinical response	—	—	0.668	—	—	0.549
Partial response	3 (27.3%)	1 (11.1%)		2 (28.6%)	1 (14.3%)	
Stable disease	4 (36.4%)	4 (44.4%)		3 (42.9%)	2 (28.6%)	
Progressive disease	4 (36.4%)	4 (44.4%)		2 (28.6%)	4 (57.1%)	

PFS (day)^a^	102.0 (43.0; 172.5)	44.0 (42.0; 76.0)	0.332	130.0 (74.0; 166.0)	43.0 (42.0; 45.0)	0.036

PD-L1 expression^b^	—	—	0.050	—	—	0.110
Negative	1 (9.1%)	4 (50.0%)		1 (14.3%)	4 (57.1%)	
>0%, ≤50%	3 (27.3%)	3 (37.5%)		1 (14.3%)	1 (14.3%)	
>50%	7 (63.6%)	1 (12.5%)		5 (71.4%)	1 (14.3%)	

EGFR mutation	—	—	0.115	—	—	0.193
Positive	0 (0.0%)	3 (33.3%)		0 (0.0%)	3 (42.9%)	
Negative	7 (63.6%)	4 (44.4%)		7 (100.0%)	4 (57.1%)	
Not done	4 (36.4%)	2 (22.2%)		—	—	

ALK mutation	—	—	0.621	—	—	0.368
Positive	1 (9.1%)	0 (0.0%)		1 (14.3%)	0 (0.0%)	
Negative	6 (54.5%)	6 (66.7%)		6 (85.7%)	6 (85.7%)	
Not done	4 (36.4%)	3 (33.3%)		0 (0.0%)	1 (14.3%)	

KRAS mutation	—	—	0.638	—	—	0.247
Positive	2 (18.2%)	1 (11.1%)		2 (28.6%)	1 (14.3%)	
Negative	4 (36.4%)	2 (22.2%)		4 (57.1%)	2 (28.6%)	
Not done	5 (45.5%)	6 (66.7%)		1 (14.3%)	4 (57.1%)	

^a^Data of 5 patients whose ICI treatment was not finished (ongoing) were excluded. ^b^Missing data (not done) of one patient were excluded for the analysis.

**Table 2:** Cytokines and immune profiling of 10 patients with non-cold or cold type.

	Non-cold (*n* = 5)	Cold (*n* = 5)	*p* value
Age (year)	55.6 ± 12.9	68.8 ± 8.3	0.091

Sex	—	—	0.168
Female	0 (0.0%)	3 (60.0%)	
Male	5 (100.0%)	2 (40.0%)	

Cytokine (pg/mL)			
IL-6 (*n* = 9)	3.8 ± 4.0	9.1 ± 7.4	0.208
IL-8 (*n* = 10)	10.1 ± 3.4	25.9 ± 23.5	0.208
IL-10 (*n* = 10)	0.6 ± 0.3	0.8 ± 0.3	0.425
IL-22 (*n* = 8)	1.2 ± 0.5	1.3 ± 0.7	0.829

Immune profiling (% intact live cells)			
Lymphocytes	73.1 ± 2.2	75.6 ± 5.4	0.379
CD3 T-cells	50.3 ± 4.5	46.0 ± 12.4	0.480
CD8 T-cells	21.0 ± 3.5	16.8 ± 4.1	0.113
Naïve	3.5 ± 2.7	1.9 ± 1.4	0.293
Central memory	0.6 ± 0.9	0.8 ± 0.7	0.719
Effector memory	3.8 ± 0.8	6.2 ± 1.6	0.015
Terminal effector	13.2 ± 1.9	7.9 ± 2.4	0.005
CD4 T-cells	25.8 ± 5.9	26.7 ± 9.3	0.860
Naïve	8.3 ± 7.0	3.8 ± 1.7	0.227
Central memory	5.4 ± 1.0	7.1 ± 3.4	0.317
Effector memory	4.2 ± 1.6	6.5 ± 1.9	0.081
Terminal effector	7.9 ± 3.2	9.3 ± 4.1	0.560
Gamma delta T-cells	3.1 ± 2.0	1.9 ± 0.6	0.273
MAIT & NKT CD4 cells	0.4 ± 0.2	0.6 ± 0.6	0.543
B cells	6.4 ± 3.1	6.9 ± 3.0	0.808
Naïve	5.6 ± 3.1	6.1 ± 3.1	0.806
Memory	0.6 ± 0.1	0.6 ± 0.2	0.985
Plasma blasts	0.2 ± 0.1	0.1 ± 0.1	0.832
NK cells	16.4 ± 1.7	22.7 ± 8.1	0.156
Early NK	2.5 ± 0.8	3.3 ± 1.2	0.264
Late NK	13.9 ± 1.0	19.5 ± 7.4	0.169
Monocytes	15.8 ± 2.9	15.2 ± 2.7	0.737
Classical	13.5 ± 2.8	13.0 ± 2.5	0.760
Transitional	1.6 ± 0.7	1.6 ± 0.6	0.851
Nonclassical	0.6 ± 0.6	0.6 ± 0.6	0.967
Dendritic cells	1.3 ± 0.2	1.1 ± 0.5	0.446
pDC	0.3 ± 0.1	0.3 ± 0.1	0.247
mDC	1.0 ± 0.2	0.9 ± 0.4	0.585
Granulocytes	1.0 ± 0.6	1.0 ± 1.3	0.998
Neutrophils	0.4 ± 0.4	0.2 ± 0.1	0.190
Basophils	0.3 ± 0.3	0.6 ± 1.0	0.630
Eosinophils	0.0 ± 0.0	0.0 ± 0.0	0.242
CD66b neutrophils	0.3 ± 0.1	0.3 ± 0.2	0.876

**Table 3:** Clinical characteristics of 20 patients classified by the cluster analysis.

	Group A (*n* = 15)	Group B (*n* = 5)	*p* value
Age (year)	60.5 ± 11.1	63.6 ± 9.2	0.577

Sex	—	—	0.011
Female	0 (0.0%)	3 (60.0%)	
Male	15 (100.0%)	2 (40.0%)	

Cold syndrome (ZHENG) symptoms^a^			
I have an aversion to cold	3.3 ± 1.2	5.0 ± 0.0	<0.001
I need to stay very warm	3.5 ± 1.0	5.0 ± 0.0	<0.001
I have experienced coldness in the abdomen	2.5 ± 1.4	4.0 ± 1.4	0.059
I feel coldness in my hands and feet	2.8 ± 1.4	3.0 ± 1.4	0.788
I have had painful cold sensations in my body	2.7 ± 1.3	4.2 ± 1.8	0.056
My face looks pale	1.5 ± 0.6	3.6 ± 1.7	0.045
I cannot drink cold water	2.9 ± 1.4	5.0 ± 0.0	<0.001
I urinate colorless urine	3.2 ± 1.0	3.2 ± 1.6	0.999

Heat syndrome (ZHENG) symptoms^a^			
I like a cool feeling	3.5 ± 1.0	1.4 ± 0.5	<0.001
I do not like hot or warm atmosphere	2.4 ± 1.0	1.4 ± 0.5	0.047
I have hot or fever sensation in my body	2.9 ± 1.3	1.8 ± 1.8	0.147
I have burning sensation in my body	1.8 ± 1.1	2.2 ± 1.6	0.537
My face looks red or eyes are blood shot	1.8 ± 1.0	1.6 ± 0.9	0.700
I like to drink cold water	3.6 ± 1.1	1.2 ± 0.4	<0.001
My breath has been hot	2.1 ± 1.0	1.4 ± 0.9	0.160

Non-cold/cold type	—	—	0.020
Non-cold type	11 (73.3%)	0 (0.0%)	
Cold type	4 (26.7%)	5 (100.0%)	

PD-L1^b^	—	—	0.051
Negative	2 (14.3%)	3 (60.0%)	
>0%, ≤50%	4 (28.6%)	2 (40.0%)	
>50%	8 (57.1%)	0 (0.0%)	

^a^ Each item is measured as a 5-point Likert score ranging (1 to 5).

## Data Availability

The data used to support the findings of this study are available from the corresponding author upon request.

## Supplementary Materials

Supplementary Materials Table S1: demographic and clinical characteristics of 12 patients with cold or heat type. Table S2: demographic and clinical characteristics of 20 patients with heat or non-heat type. Table S3: demographic and clinical characteristics of 20 patients classified by cluster analysis.
