# Supplementary material for: A Pilot Study of Whether the Cold-Heat Syndrome Type is Associated with Treatment Response and Immune Status in Patients with Non-Small Cell Lung Cancer
Source: Evid Based Complement Alternat Med. 2021 Jun 22;2021:9920469. doi: 10.1155/2021/9920469 (PMC8241512; doi:10.1155/2021/9920469)
Supplement: Supplementary Materials — Table S1: demographic and clinical characteristics of 12 patients with cold or heat type. Table S2: demographic and clinical characteristics of 20 patients with heat or non-heat type. Table S3: demographic and clinical characteristics of 20 patients classified by cluster analysis. [file 9920469.f1.docx]

**Supplementary Table 1**. Demographic and clinical characteristics of 12 patients with cold or heat type.

|  | Heat  (n=4) | Cold  (n=8) | p-value |
| --- | --- | --- | --- |
| **Age** (year) | 57.8 ± 3.3 | 61.5 ± 9.4 | 0.465 |
| **Sex** |  |  | 0.480 |
| Female | 0 ( 0.0 %) | 3 (37.5 %) |  |
| Male | 4 (100.0 %) | 5 (62.5 %) |  |
| **Smoking status** |  |  | 0.165 |
| Current | 3 (75.0 %) | 2 (25.0 %) |  |
| Ex-smoker | 1 (25.0 %) | 2 (25.0 %) |  |
| Non-smoker | 0 ( 0.0 %) | 4 (50.0 %) |  |
| **Histologic type** |  |  | 0.480 |
| Adenocarcinoma | 2 (50.0 %) | 7 (87.5 %) |  |
| Squamous | 2 (50.0 %) | 1 (12.5 %) |  |
| **Drug** |  |  | 0.080 |
| Pembrolizumab | 2 (50.0 %) | 1 (12.5 %) |  |
| Nivolumab | 1 (25.0 %) | 0 ( 0.0 %) |  |
| Atezolizumab | 1 (25.0 %) | 7 (87.5 %) |  |
| **Line of therapy** |  |  | 0.038 |
| 2^nd^ line therapy | 4 (100.0 %) | 1 (12.5 %) |  |
| 3^rd^ line therapy | 0 ( 0.0 %) | 2 (25.0 %) |  |
| 4^th^ line therapy | 0 ( 0.0 %) | 4 (50.0 %) |  |
| 7^th^ line therapy | 0 ( 0.0 %) | 1 (12.5 %) |  |
| **No. of cycle** ^a^ | 3.7 ± 3.1 | 4.3 ± 2.5 | 0.735 |
| Ongoing | 1 (25.0 %) | 2 (25.0 %) |  |
| **Clinical response** |  |  | 0.829 |
| Partial Response | 1 (25.0 %) | 1 (12.5 %) |  |
| Stable Disease | 1 (25.0 %) | 3 (37.5 %) |  |
| Progressive Disease | 2 (50.0 %) | 4 (50.0 %) |  |
| **PFS** (day) ^a^ | 37.0 [29.0; 83.5] | 43.0 [42.0; 103.0] | 0.368 |
| **PD-L1** ^b^ |  |  | 0.231 |
| Negative | 0 ( 0.0 %) | 3 (37.5 %) |  |
| >0 %, ≤50 % | 1 (25.0 %) | 2 (25.0 %) |  |
| >50 % | 3 (75.0 %) | 2 (25.0 %) |  |
| **EGFR** |  |  | 0.223 |
| Positive | 0 ( 0.0 %) | 3 (37.5 %) |  |
| Negative | 2 (50.0 %) | 4 (50.0 %) |  |
| Not done | 2 (50.0 %) | 1 (12.5 %) |  |
| **ALK** |  |  | 0.829 |
| Negative | 2 (50.0 %) | 6 (75.0 %) |  |
| Not done | 2 (50.0 %) | 2 (25.0 %) |  |
| **KRAS** |  |  | 0.519 |
| Positive | 1 (25.0 %) | 1 (12.5 %) |  |
| Negative | 0 ( 0.0 %) | 2 (25.0 %) |  |
| Not done | 3 (75.0 %) | 5 (62.5 %) |  |

^a^ Data of 4 patients whose ICI treatment was not finished (ongoing) were excluded.

^b^ Missing data (Not done) of one patient were excluded for the analysis.

**Supplementary Table 2**. Demographic and clinical characteristics of 20 patients with heat or non-heat type.

|  | Heat  (n=5) | Non-heat  (n=15) | p-value |
| --- | --- | --- | --- |
| **Age** (year) | 61.8 ± 9.5 | 61.1 ± 11.1 | 0.897 |
| **Sex** |  |  | 0.718 |
| Female | 0 ( 0.0 %) | 3 (20.0 %) |  |
| Male | 5 (100.0 %) | 12 (80.0 %) |  |
| **Smoking status** |  |  | 0.298 |
| Current | 4 (80.0 %) | 7 (46.7 %) |  |
| Ex-smoker | 1 (20.0 %) | 3 (20.0 %) |  |
| Non-smoker | 0 ( 0.0 %) | 5 (33.3 %) |  |
| **Histologic type** |  |  | 0.260 |
| Adenocarcinoma | 2 (40.0 %) | 12 (80.0 %) |  |
| Squamous | 3 (60.0 %) | 3 (20.0 %) |  |
| **Drug** |  |  | 0.837 |
| Pembrolizumab | 2 (40.0 %) | 4 (26.7 %) |  |
| Nivolumab | 1 (20.0 %) | 3 (20.0 %) |  |
| Atezolizumab | 2 (40.0 %) | 8 (53.3 %) |  |
| **Line of therapy** |  |  | 0.141 |
| 2^nd^ line therapy | 5 (100.0 %) | 6 (40.0 %) |  |
| 3^rd^ line therapy | 0 ( 0.0 %) | 3 (20.0 %) |  |
| 4^th^ line therapy | 0 ( 0.0 %) | 5 (33.3 %) |  |
| 7^th^ line therapy | 0 ( 0.0 %) | 1 (6.7 %) |  |
| **No. of cycle** ^a^ | 3.8 ± 2.5 | 5.8 ± 5.0 | 0.453 |
| Ongoing | 1 (20.0 %) | 3 (20.0 %) |  |
| **Clinical response** |  |  | 0.999 |
| Partial Response | 1 (20.0 %) | 3 (20.0 %) |  |
| Stable Disease | 2 (40.0 %) | 6 (40.0 %) |  |
| Progressive Disease | 2 (40.0 %) | 6 (40.0 %) |  |
| **PFS** (day) ^a^ | 56.5 [29.0; 103.0] | 61.5 [43.0; 166.0] | 0.357 |
| **PD-L1** ^b^ |  |  | 0.252 |
| Negative | 0 ( 0.0 %) | 5 (33.3 %) |  |
| >0 %, ≤50 % | 1 (20.0 %) | 3 (20.0 %) |  |
| >50 % | 4 (80.0 %) | 6 (40.0 %) |  |
| **EGFR** |  |  | 0.195 |
| Positive | 0 ( 0.0 %) | 3 (20.0 %) |  |
| Negative | 2 (40.0 %) | 9 (60.0 %) |  |
| Not done | 3 (60.0 %) | 3 (20.0 %) |  |
| **ALK** |  |  | 0.374 |
| Negative | 0 ( 0.0 %) | 1 ( 6.7 %) |  |
| Not done | 2 (40.0 %) | 10 (66.7 %) |  |
| **KRAS** |  |  | 0.238 |
| Positive | 1 (20.0 %) | 2 (13.3 %) |  |
| Negative | 0 ( 0.0 %) | 6 (40.0 %) |  |
| Not done | 4 (80.0 %) | 7 (46.7 %) |  |

^a^ Data of 4 patients whose ICI treatment was not finished (ongoing) were excluded.

^b^ Missing data (Not done) of one patient were excluded for the analysis.

**Supplementary Table 3**. Demographic and clinical characteristics of 20 patients classified by cluster analysis.

|  | Group A  (n=15) | Group B  (n=5) | p-value |
| --- | --- | --- | --- |
| **Age** (year) | 60.5 ± 11.1 | 63.6 ± 9.2 | 0.577 |
| **Sex** |  |  | 0.011 |
| Female | 0 ( 0.0 %) | 3 (60.0 %) |  |
| Male | 15 (100.0 %) | 2 (40.0 %) |  |
| **Smoking status** |  |  | 0.093 |
| Current | 10 (66.7 %) | 1 (20.0 %) |  |
| Ex-smoker | 3 (20.0 %) | 1 (20.0 %) |  |
| Non-smoker | 2 (13.3 %) | 3 (60.0 %) |  |
| **Histologic type** |  |  | 0.999 |
| Adenocarcinoma | 10 (66.7 %) | 4 (80.0 %) |  |
| Squamous | 5 (33.3 %) | 1 (20.0 %) |  |
| **Drug** |  |  | 0.036 |
| Pembrolizumab | 6 (40.0 %) | 0 ( 0.0 %) |  |
| Nivolumab | 4 (26.7 %) | 0 ( 0.0 %) |  |
| Atezolizumab | 5 (33.3 %) | 5 (100.0 %) |  |
| **Line of therapy** |  |  | 0.062 |
| 2^nd^ line therapy | 10 (66.7 %) | 1 (20.0 %) |  |
| 3^rd^ line therapy | 1 (6.7 %) | 2 (40.0 %) |  |
| 4^th^ line therapy | 4 (26.7 %) | 1 (20.0 %) |  |
| 7^th^ line therapy | 0 (0.0 %) | 1 (20.0 %) |  |
| **No. of cycle** ^a^ | 6.3 ± 5.2 | 3.2 ± 1.3 | 0.085 |
| Ongoing | 5 (33.3 %) | 0 ( 0.0 %) |  |
| **Clinical response** |  |  | 0.368 |
| Partial Response | 5 (33.3 %) | 3 (60.0 %) |  |
| Stable Disease | 4 (26.7 %) | 0 ( 0.0 %) |  |
| Progressive Disease | 6 (40.0 %) | 2 (40.0 %) |  |
| **PFS** (day) ^a^ | 75.0 [42.0; 166.0] | 44.0 [42.5; 74.0] | 0.479 |
| **PD-L1** ^b^ |  |  | 0.051 |
| Negative | 2 (14.3 %) | 3 (60.0 %) |  |
| >0 %, ≤50 % | 4 (28.6 %) | 2 (40.0 %) |  |
| >50 % | 8 (57.1 %) | 0 ( 0.0 %) |  |
| **EGFR** |  |  | 0.005 |
| Positive | 0 ( 0.0 %) | 3 (60.0 %) |  |
| Negative | 10 (66.7 %) | 1 (20.0 %) |  |
| Not done | 5 (33.3 %) | 1 (20.0 %) |  |
| **ALK** |  |  | 0.827 |
| Negative | 5 (33.3 %) | 2 (40.0 %) |  |
| Not done | 9 (60.0 %) | 3 (60.0 %) |  |
| **KRAS** |  |  | 0.372 |
| Positive | 7 (46.7 %) | 4 (80.0 %) |  |
| Negative | 5 (33.3 %) | 1 (20.0 %) |  |
| Not done | 3 (20.0 %) | 0 ( 0.0 %) |  |

^a^ Data of 7 patients whose ICI treatment was not finished (ongoing) were excluded.

^b^ Missing data (Not done) of one patient were excluded for the analysis.
